# The study on biological activities of silver nanoparticles produced via green synthesis method using *Salvia officinalis* and *Thymus vulgaris*

**DOI:** 10.55730/1300-0527.3448

**Published:** 2022-05-09

**Authors:** Ömer ÖDEMİŞ, Sadin ÖZDEMİR, Serpil GONCA, Ali ARSLANTAŞ, Mehmet Salih AĞIRTAŞ

**Affiliations:** 1 Department of Chemistry, Faculty of Science Faculty of Science, Van Yüzüncü Yıl University, Van, Turkey; 2 Food Processing Program, Technical Science Vocational School, Mersin University, Mersin, Turkey; 3 Department of Pharmaceutical Microbiology, Faculty of Pharmacy, University of Mersin, Turkey; 4 Department of Biomedical Engineering, School of Engineering and Architecture, İzmir Bakırçay University, İzmir, Turkey

**Keywords:** Green synthesized, AgNPs, *Salvia officinalis*, *Thymus vulgaris*, biological properties

## Abstract

In the present study, Ag nanoparticles (AgNPs) were synthesized from *Salvia officinalis* and *Thymus vulgaris*, known as phytotherapy plants. The obtained silver nanoparticles were characterized using SEM, XRD, FTIR, and UV/Vis spectra. The antioxidant capacities of *Salvia officinalis-*mediated AgNP (SO-AgNP) and *Thymus vulgaris*-mediated AgNP (TV-AgNP) were analyzed in vitro using 1,1-diphenyl-2-picrylhydrazyl and iron chelating activity assays. DPPH activities were 83.74% and 57.17% for SO-AgNP and TV-AgNP at concentration 200 mg/L, respectively. Both green synthesized AgNPs exhibited good iron chelating activity. In addition, the DNA cleavage activities of SO-AgNPs and TV-AgNP were investigated with agarose gel electrophoresis technique. SO-AgNPs and TV-AgNP showed single-strand DNA cleavage activity. AgNPs showed that the SO-AgNP and TV-AgNp were effective against bacteria and fungi, and antimicrobic activities were assessed as minimal inhibition concentration (MIC). Remarkably, green synthesized AgNPs showed highly effective cell viability and biofilm inhibition effect. AgNPs also demonstrated slightly antimicrobial photodynamic activity after LED irradiation.

## 1. Introduction

Nanotechnology is a significant area in sciences, which is interested in the synthesis of very tiny materials ranging from 1 to 100 nm [1]. The nanoparticles have incomparable features thanks to which they play significant roles in antimicrobial, biomedicine, biosensor, and medical applications [2]. One of the well-known metallic nanoparticles are silver particles which earned great interest in the nanotechnology field and nanomedicine fields [3], which perform significant roles in nanomedicine due to their antimicrobial activities [4–8]. Nowadays, many research studies are investigating the nanoparticle effect. It is seen as an effective alternative to silver for catalysts, antiseptic agents, medicine, and cosmetics [4]. Obtaining this element with an environmentalist approach known as green synthesis and investigating its biological properties are current study topics [9]. Factors such as nontoxicity can exhibit superior potential properties such as nano-drug delivery and effective antioxidants. Nano-sized particles have drawn great attention in biomedical technology [10,11]. AgNPs have gained concern because of their small particle size, large surface area, and electrochemical properties [12]. AgNPs have a high capacity, such as antimicrobial [13], antioxidant [14], antibiofilm [15], anticancer [16], and antiinflammatories [17].

Firstly, *Salvia officinalis* and *Thymus vulgaris*, known as phytotherapy plant AgNPs, were synthesized in this study. These products were then characterized with spectroscopic methods such as SEM, XRD, FTIR, and UV-Vis spectra. After the product was obtained, DPPH activity, ferrous ion chelating activity, antimicrobial activity, DNA splitting ability, bacterial viability test, antimicrobial photodynamic treatment with green synthesized AgNPs, and biofilm inhibition activity properties of these two nanoparticles were investigated.

## 2. Experimental

### 2.1. Materials

*Salvia officinalis* and *Thymus vulgaris* plants were purchased from herbalists in dried-ready form (Turkey). Silver nitrate, 1-diphenyl-2-picrylhydrazyl, ascorbic acid, Trolox, Iron(II) chloride was purchased from Sigma Company. Whatman No.1 and No.2 filter papers were used for filtering. Electronic absorption of silver nanoparticles was determined by PhotoLab 7600 UV-VIS spectrophotometer (Van Yüzüncü Yıl University, Turkey). ZEISS Sigma 300 SEM instrument was used for surface morphology (Atatürk University, Turkey). PANalytical Empyrean XRD was used for the qualitative identification of nanoparticles (Atatürk University, Turkey). A Thermo Scientific FT-IR spectrophotometer was used for characterization of nanoparticles (Van Yüzüncü Yıl University, Turkey).

### 2.2. Preparation of the plant extracts

Twenty grams of *Salvia officinalis* (SO) and 20 grams of *Thymus vulgaris* (TV) plants were taken separately and washed with pure water. It was boiled in 500 mL of distilled water on a magnetic stirrer at 80 °C for 30 min. After the extraction was completed, the obtained extracts were permitted to cool down to 25 °C. Then, the solid materials were filtered using Whatman No.1 filter paper; after filtration, they were stored at +4 °C in the dark for biosynthesis processes.

### 2.3. Synthesis of silver nanoparticles

**Figure 1:**
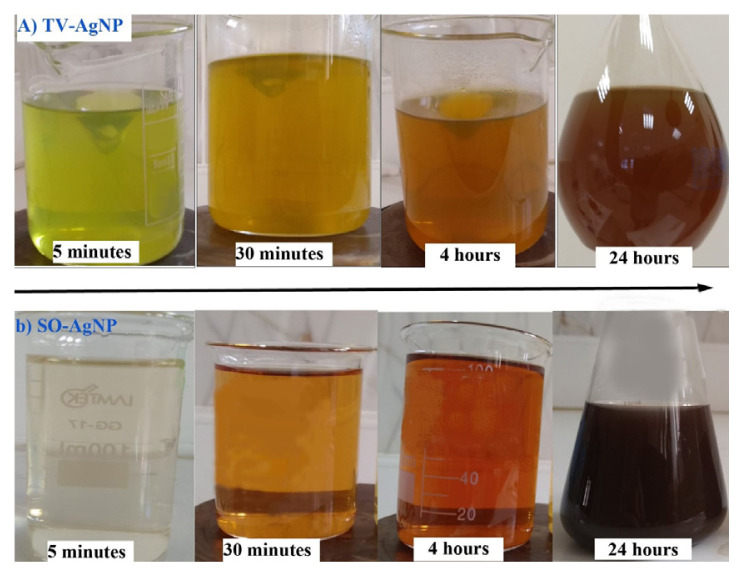
(a) The color change observed within 24 h when the *s*ilver nitrate solution is mixed with TV extract. (b) Color change observed within 24 h when SO extract is mixed with silver nitrate solution.

Fifty milliliters of SO and TV extracts were added to the previously prepared 500 mL of 1mM AgNO_3_ solutions at the ratio (10:1), and color change was observed depending on the formation of silver nanoparticles. The reduction of Ag^+^ ions to Ag^o^ was completed by turning the mixture dark brown as indicated in the literature (Figure 1) [18].

The resulting dark brown mixture was centrifuged at 4500 rpm for 90 min. The supernatant was removed, and the residue solid was washed with distilled water, followed by Whatman No 1. It was filtered using two filter papers. The AgNPs were powdered after being kept in an oven at 37 °C for 48 h and stored to determine their characteristic properties. Experimental methodology of DPPH activity [19], ferrous ion chelating activity [20], antimicrobial activity, DNA splitting ability, bacterial viability test, antimicrobial photodynamic therapy with AgNPs, and biofilm inhibition activity are given in the supplementary information section.

## 3. Results and discussion

### 3.1. Silver nanoparticles with UV VIS spectrophotometer characterization

**Figure 2:**
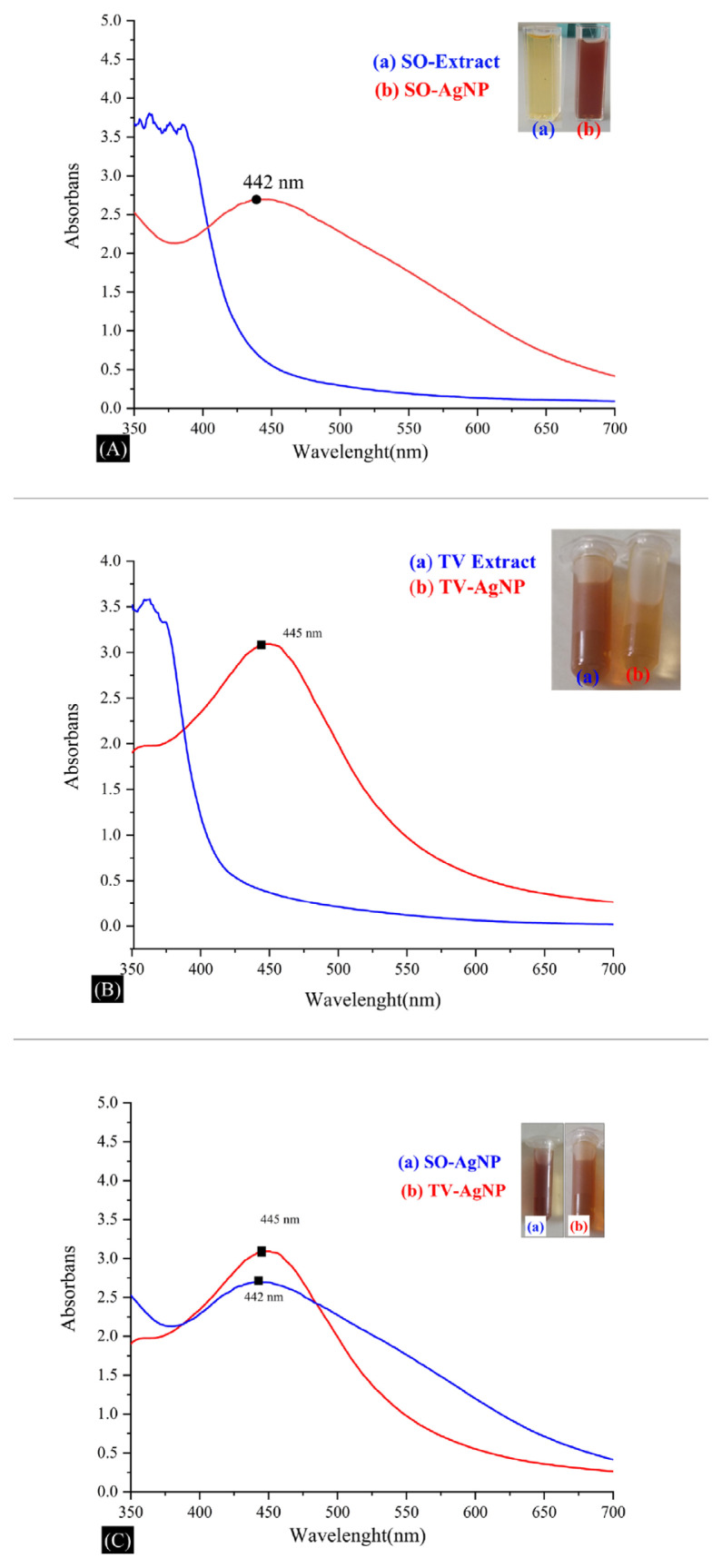
A) UV/Vis absorption spectra of synthesized TV-AgNPs. a) TV-AgNP solution, b) TV extract, B) UV/Vis absorption spectra of synthesized SO-AgNPs. a) SO extract, b) SO-AgNP solution. C) Comparison of UV/Vis absorption spectra of synthesized SO-AgNPs and TV-AgNPs.

The absorption of Ag nanoparticles in the range of 300–700 nm was estimated using a UV/Vis spectrophotometer. A strong and broad surface plasmon peak was observed at 445 nm for AgNPs prepared using *Thymus vulgaris* extract and 442nm for AgNPs prepared using *Salvia officinalis* (Figure 2). As can be seen in the literature, the accuracy of the peaks was confirmed [21].

### 3.2. The XRD analysis

**Figure 3:**
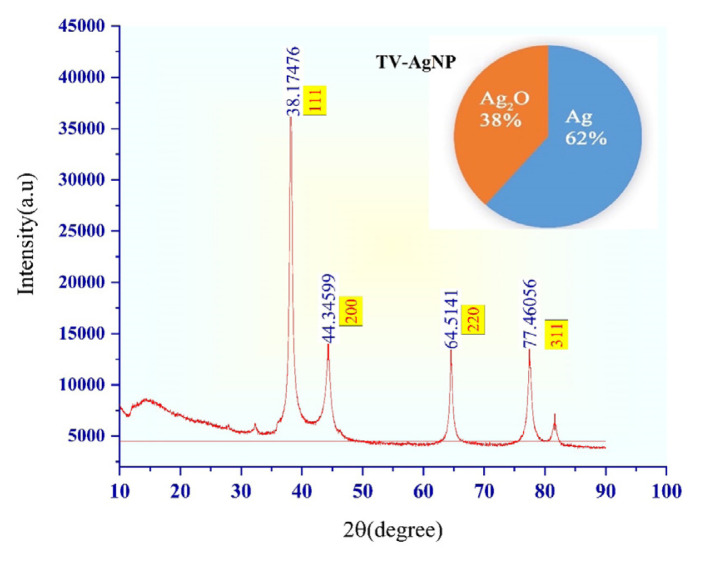
X-ray diffraction pattern of TV-Ag nanoparticles and percentage of Ag.

**Figure 4:**
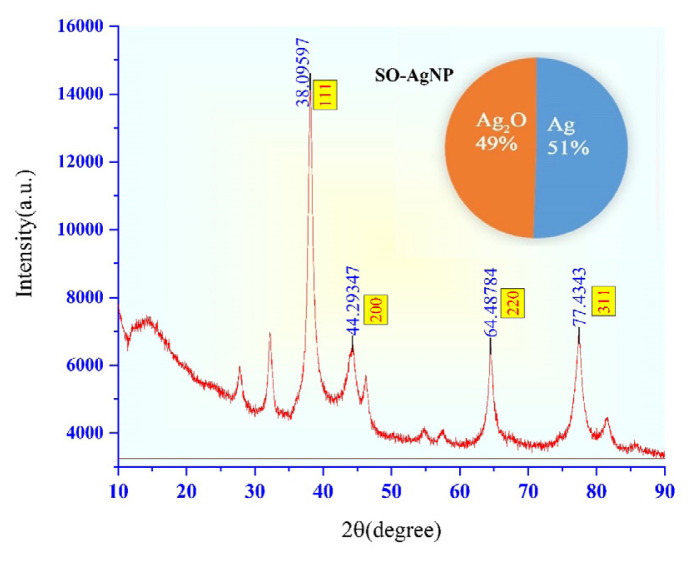
X-ray diffraction pattern of SO-Ag nanoparticles and percentage of Ag.

Information about the geometric arrangement of the atoms of the crystals in AgNPs and the distance between them are obtained by this method. Therefore, X-ray diffraction (XRD) has been used because it is a practical and convenient method for the qualitative identification of crystalline compounds. Analysis of the XRD phase and crystal structure analysis of plant-synthesized AgNPs are shown in Figures 3 and 4. The percentage of crystallinity of TV-AgNPs synthesized with *Thymus vulgaris* plant extract, the ratio of the sum of the areas of the peaks with specific peaks to the sum of all areas was found to be 62% (Figure 3.). Similarly, the percentage of crystallinity of SO-AgNPs was found to be 51% (Figure 4).

In the XRD analysis of TV-AgNPs, the values corresponding to the value (Figure 3) (38.18°, 44.35°, 64.51°, and 77.46°) were calculated. It has been determined that the nanoparticles formed are elemental Ag and in the spherical crystal structure. By calculating the XRD data with the Debye-Scherrer in equality, the particle mean size was 50.4 nm.

While the same procedure is performed in SO-AgNPs (Figure 4), in XRD analysis, values corresponding to those of Figure 4 (38.09°, 44.29°, 64.49°, 77.43°, 81.64°) were calculated. It was found that the nanoparticles formed were also in the crystalline and spherical crystal structure, and the average particle size was 42.3 nm as a result of calculating these data with the Debye-Scherrer equation [22].

### 3.3. Scanning electron microscopy (SEM) and EDX study of AgNPs

**Figure 5:**
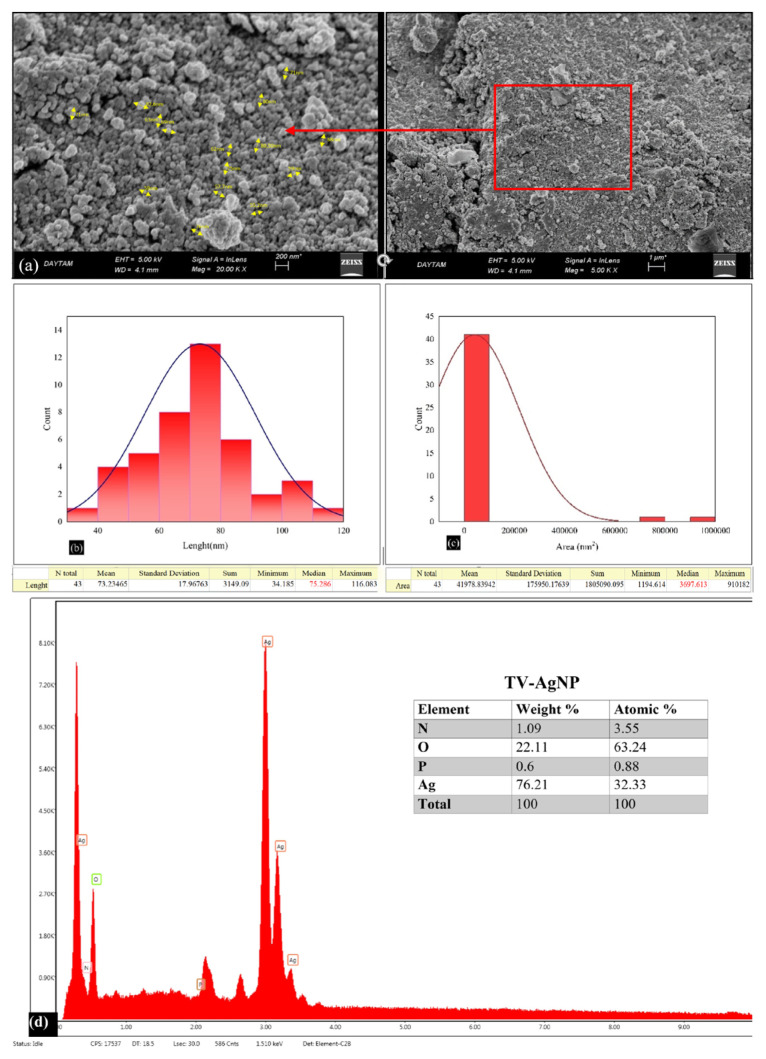
a) SEM images showing the morphological structures of TV-AgNPs, b) Average molecular sizes of TV-AgNPs, c) Average molecular areas of TV-AgNPs, and d) EDX spectrum and element dispersions.

**Figure 6:**
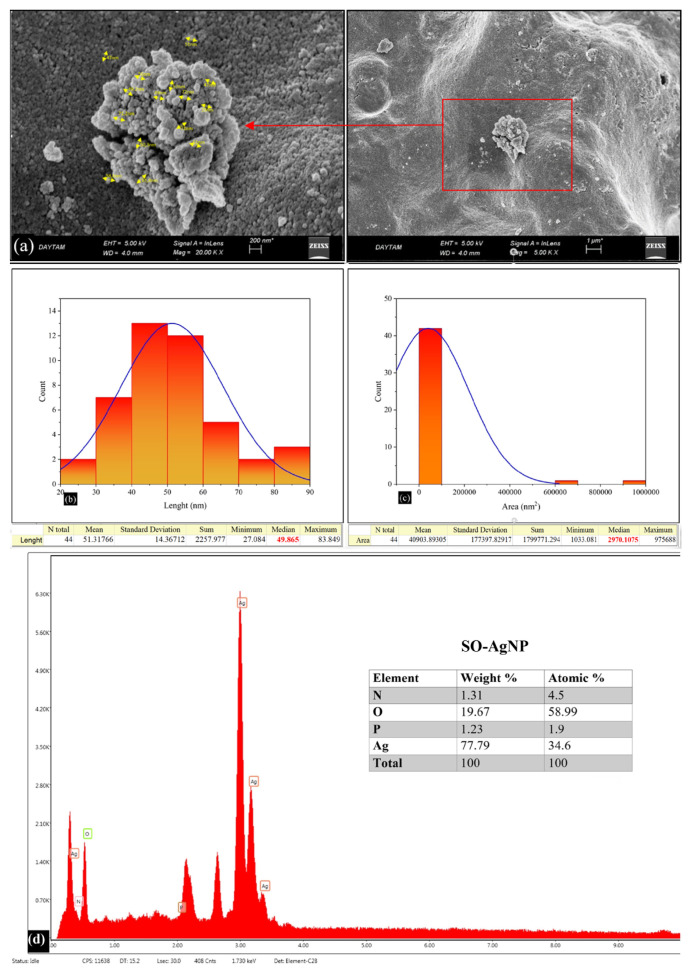
a) SEM images showing the morphological structures of SO-AgNPs, b) Average molecular sizes of SO-AgNPs, c) Average molecular areas of SO-AgNPs, and d) EDX spectrum and element dispersions.

In the morphology of nanoparticles, SEM analyses are used quite effectively for the size and shape of the nanomaterial. SEM images of AgNPs prepared in this study are given in Figures 5 and 6. When the formation and morphological dimensions of TV-AgNPs and SO-AgNPs were examined in the SEM study, it was determined that the distance between particles and their average size were 75.285 nm and 49.865 nm, respectively. AgNPs composed of *Thymus vulgaris* (TV) and *Salvia officinalis* plant extracts appear to have similar spherical structures.

### 3.4. The infrared spectroscopy study

FT-IR spectroscopy was applied to determine bond vibrations in the structure of molecules. The broad bands appeared at ~3437cm^−1^ and 3508 cm^−1^ in spectra due to O-H stretching vibration. The band that appeared at 1732 cm^−1^ is referred to as C=O stretching vibration for TV-AgNPs. The band that appeared at 1529 cm^−1^ is attributed to C=C vibration of stretching for SO-AgNP. The bands at 1205 cm^−1^ and 1215 cm^−1^ are referred to C-O aromatic vibrations, and this indicates that it is due to the vibrations of the groups that may exist between the silver and the extracts.

### 3.5. DPPH radical scavenging activity

**Figure 7:**
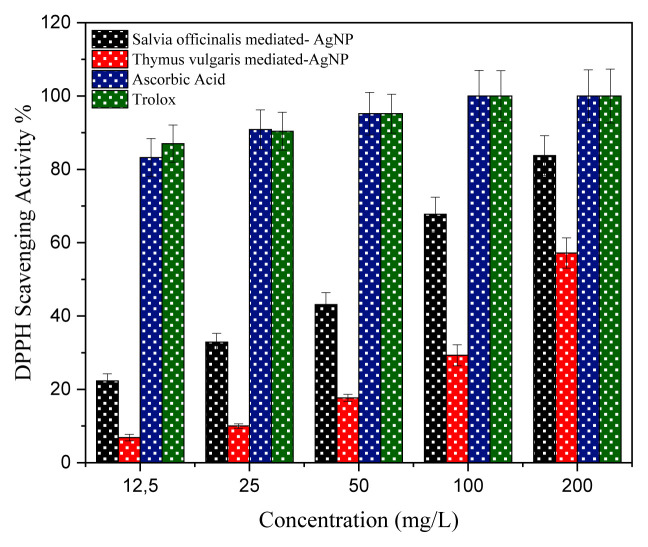
DPPH scavenging activity.

Antioxidants are of particular interest because they defend the human body against radicals and oxidative stress-related ailments. Briefly, 1-diphenyl-2-picrylhydrazyl (DPPH) is a stable free radical and is well known for its role in reducing electron or hydrogen acceptance from donors [23,24]. In addition, DPPH has dark purple color in an aqueous solution with the highest absorption peak of 517 nm. When DPPH is reduced, the purple color is shifted to yellow, diminishing absorbance at 517 nm. The DPPH scavenging activity of the newly synthesized SO-AgNp and TV-AgNp was studied based on the color change of the DPPH solution. The graph below shows the results of the DPPH scavenging activities of the synthesized nanoparticles and standards (Figure 7). According to the results, the DPPH activity of the synthesized nanoparticles was concentration-dependent. Similar findings were also observed for AgNPs synthesized of the plant *Pyrus betulifolia Bunge* [11] and *Cleome viscosa* extract [23]. As shown in Figure 7, antioxidant activity results of SO-AgNp were better than those of TV-AgNp. When the compound concentration increased from 12.5 mg/L to 100 mg/L, the scavenging activities of SO-AgNp and TV-AgNp were increased from 22.30% to 67.76% and from 6.84% to 29.30%, respectively. Moreover, DPPH activities of SO-AgNp, TV-AgNp, Ascorbic acid, and Trolox at a concentration of 200 mg/L were 83.74%, 57.17%, 100%, and 100%, respectively.

Sudha et al. [22] synthesized a novel green AgNp applying *Lippia nodiflora* aerial extract and evaluated the antibacterial properties. They reported that AgNPs exhibited better antioxidant activity of 67% at 500 μg/mL. We can say that the present study results are better compared to those of the mentioned study. Moreover, there have been various studies investigating the DPPH activity of AgNp. Kumar et al. [25] synthesized AgNPs through aqueous leaf extract of *Polyalthia longifolia*, and they noticed that its DPPH activity at maximum concentrations was 82% at 100 mg/mL. Ravichandran et al. [26] synthesized Ag nanoparticles using *Artocarpus altilis* leaf extract. They reported that maximum DPPH activity was 79.79% at 100 μg/mL. In the study, the highest DPPH scavenging activity for SO-AgNp was detected at 83.74% at an amount of 200 mg/L. The increase in antioxidant activity can be referred to as the absorption of slightly bioactive compounds on the spherical and nano-sized silver particles [11]. The obtained results demonstrated that newly synthesized SO-AgNp could be used as an antioxidant agent after further studies.

### 3.6. The study of ferrous ion chelating property

**Figure 8:**
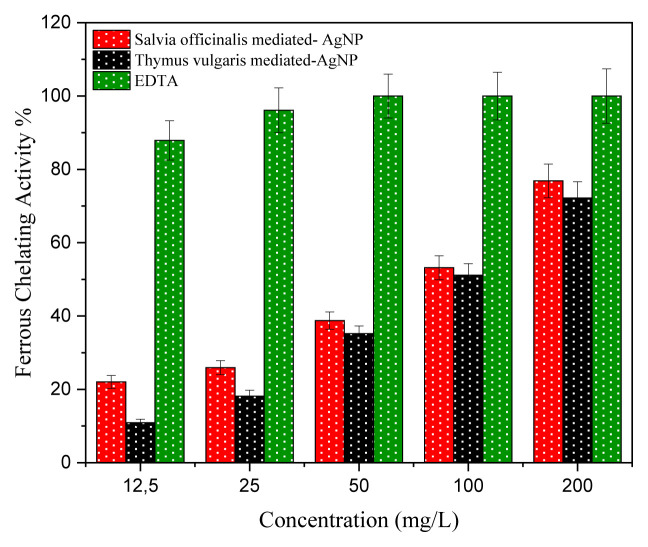
Ferrous chelating activity.

Antioxidant properties of the synthesized silver nanoparticles were also studied using the Fe^2+^ chelating technique. Antioxidants are also necessary to defend cells and bioactive macromolecules from damage through reactive oxygen species and degenerative reactions generated by free radicals [22]. The antioxidant activity results of SO-AgNP and TV-AgNP are shown in Figure 8. The green synthesized SO-AgNp and TV-AgNp displayed an excellent antioxidant activity, and chelating activity increased depending on the concentration. The results showed that the percentage inhibition of DPPH by SO-AgNP, TV-AgNP, and EDTA increased from 22.03% to 38.75%, from 10.9% to 35.18%, and from 87.9% to 100%, when concentration increased from 12.5 to 50 mg/L, respectively. SO-AgNp has better results than TV-AgNP at all concentrations, and the chelating activities of SO-AgNP and TV-AgNP were 53.15% and 51.14% at concentrations of 100 mg/L, respectively. The ferrous iron-chelating activities of silver nanoparticles were in the following order of EDTA> SO-AgNp > TV-AgNp at 200 mg/L, and they were 100%, 76.85%, and 72.18%, respectively. Kumar et al. [27] green synthesized AgNPs through leaf extract of *Holoptelea integrifolia* and investigated its antioxidant activities. They reported that AgNPs exhibited 41.18 ± 2.27% metal chelating activities. Our results have better results than the mentioned study, and these results demonstrated that newly synthesized AgNPs, mainly SO-AgNP, could be used as the chelating agent.

### 3.7. Antimicrobial activity

The antimicrobial activity of SO-AgNP and TV-AgNP was carried out through a microdilution test. The findings of antimicrobial activity are presented in Table. As seen in Table, SO-AgNP was more effective than TV-AgNP against all the microorganisms studied. MIC values of SO-AgNp and TV-AgNP were 32–256 mg/L and 64–512 mg/L respectively. Moreover, it was found that the synthesized nanoparticles were more effective against gram-positive bacteria than gram-negative bacteria. This situation can be explained as gram-negative bacteria protect themselves against antibiotics due to an outer membrane forming a permeability barrier [28]. Similar results about the bactericidal effect of green synthesized AgNPs have been introduced in some previous studies. Huong et al. [29] synthesized AgNP with the green synthesis method and reported that it exhibited more effective antimicrobial activity against gram-positive bacteria than gram-negative. However, different mechanisms have been attributed to the antibacterial activity of AgNPs. These diverse mechanisms include impairment of the bacterial cell membrane, induced ROS, impairment of DNA replication, and respiratory function [24]. *E. fecalis* was the most sensitive microorganism against synthesized nanoparticles, and MIC values were determined as 32 mg/L and 64 mg/L for SO-AgNP and TV-AgNP, respectively.

Similarly, Murugesan et al. [30] green synthesized Ag nanoparticles and studied the antimicrobial activity of AgNPs, and they reported that the most affected microorganism was *E. faecalis*. Many studies show the antimicrobial activity of green synthesis silver nanoparticles [31, 32]. This data indicates that the newly synthesized AgNPs have antimicrobial activity against gram-positive bacteria, gram-negative bacteria, and fungal strain with varying degrees of activity against several microbes.

### 3.8. The study of DNA cleavage

**Figure 9:**
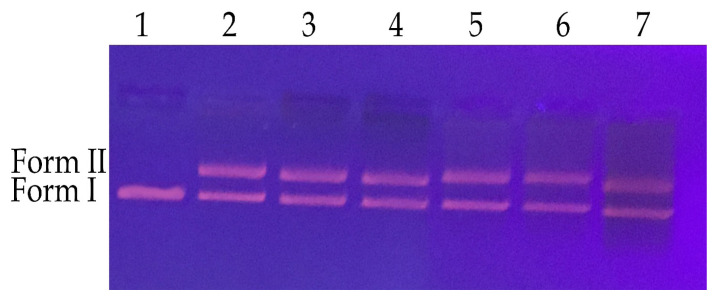
DNA cleavage property of the synthesized ApNPs a) Lane 1 represents pBR 322 DNA; Lane 2 represents pBR 322 DNA + 50 mg/L *S. officinalis*-mediated AgNP; *Lane* 3 shows pBR 322 DNA + 100 mg/L of *S. officinalis*-mediated AgNP*;* Lane 4 stands for pBR 322 DNA + 200 mg/L *S. officinalis*-mediated AgNP; Lane 5 represents pBR 322 DNA + 50 mg/L of *T. vulgaris*-mediated AgNP; Lane 6 represents pBR 322 DNA + 100 mg/L *T. vulgaris*-mediated AgNP; Lane 7 shows pBR 322 DNA + 200 mg/L *T. vulgaris*-mediated AgNP.

To explore DNA cleavage properties of recently synthesized AgNPs, *E. coli* pBR 322 plasmids DNA was used. The findings are as shown in Figure 9. As indicated in Figure 12, SO-AgNP and TV-AgNP showed single-strand DNA cleavage activity at all studied concentrations (50, 100, and 200 mg/L), and plasmid DNA was shifted from Form I to Form II in lanes 2–7. It is known that silver has a significant affinity to react with phosphorus or sulfur, including biomolecules in the cell. Thus, sulfur-including proteins in the inside cells or membrane and phosphorus including elements such as DNA are probably the preferential sites to AgNPs binding. In the present study, breaks in the DNA chain can be explained [33]. Aygün et al. [34] performed *Reishi mushroom-*mediated green synthesizing of AgNPs and studied its DNA cleavage activity. They reported that AgNPs led to single-strain DNA cleavage activity. Our results are consistent with those of the mentioned study. The study results showed that NPs acted as chemical nucleases, and after further study, they can be used as a chemical nucleases agent.

### 3.9. Microbial cell viability

**Figure 10:**
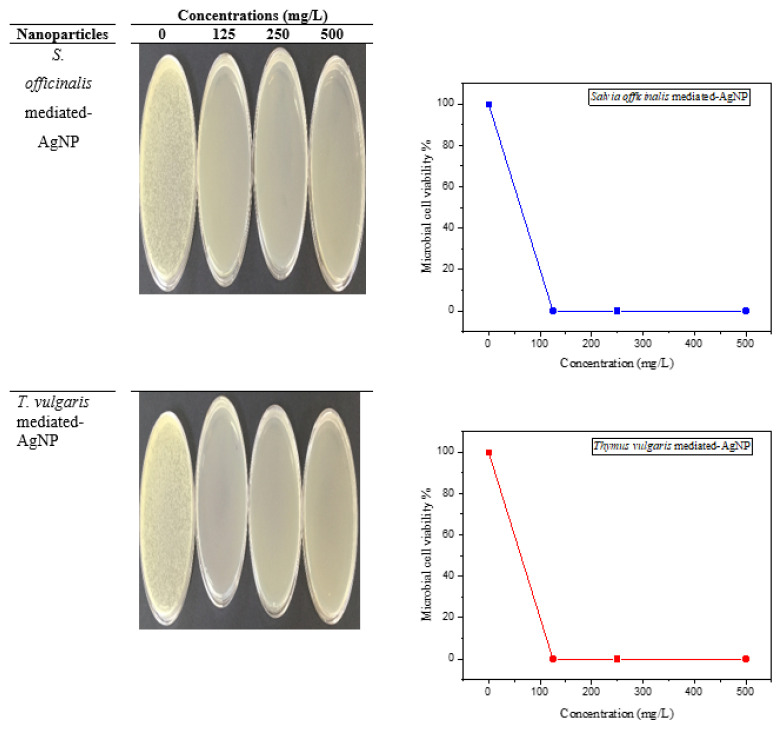
Microbial cell viability.

Silver is a precious antimicrobic agent that ascertains low toxic activity in humans and comprises diverse in vitro and in vivo implementations among the farther metals [22]. In addition to this, the antibacterial features of AgNPs have been widely admitted due to their less toxic nature than silver ions [35]. In the present work, the cell viability inhibition of green synthesized SO-AgNPs and TV-AgNPs was also studied against *E. coli*. According to our results, SO-AgNp and TV-AgNp exhibited 100% cell viability inhibition activity at all tested concentrations (125, 250, and 500 mg/L), and the findings are shown in Figure 10. The high activity of Ag nanoparticles synthesized using *Salvia officinalis* and *Thymus vulgaris* can originate from the structural changes in the membrane structure of bacterial cell wall through the action of silver nanoparticles and enhance membrane permeability, which consequently leads to bacterial cell death [22]. Kumar et al. [33] synthesized green AgNPs using *Physalis angulata* and studied its cell viability test versus *S. aureus* and *E. coli.* They proved that lack of the cell viability of *E. coli* and *S. aureus* increased steadily with extension in the incubation time. The present study is in accordance with the mentioned study. As a result, the present study suggests that the green synthesized AgNPs can be exploited more for potential biomedical applications.

### 3.10. Antimicrobial photodynamic therapy by green synthesized AgNPs

**Figure 11:**
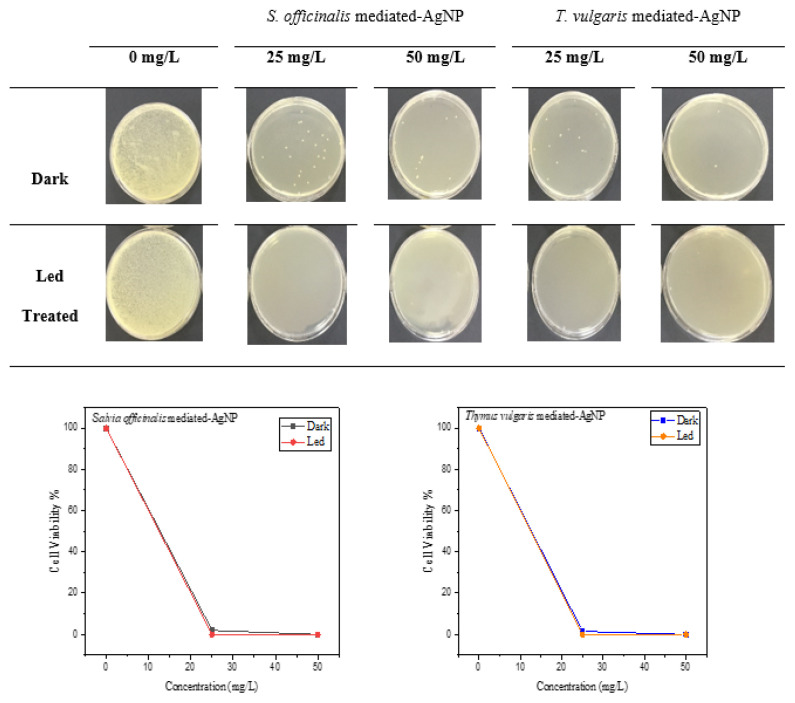
Antimicrobial photodynamic therapy of SO-AgNP and TV-AgNP.

As increasing resistance of bacterial strains against antibiotics is a big problem, searching for alternative treatments is needed. One of the options is antimicrobial photodynamic therapy (APDT). Principally, the photosensitizer is activated via the light of convenient wavelength resulting in the formation of reactive oxygen species (ROS) that are cytotoxic for the pathogen microorganisms [36]. In the present study, we also tried to study APDT of newly synthesized AgNPs against *E. coli* as a photosensitizer. The APDT results of SO-AgNP and TV-AgNP are shown in Figure 11. In the dark application, the inhibition rates for SO-AgNP and TV-AgNP at concentrations of 25 and 50 mg/L were 98%, 99.99% and 98.5%, 99.99%, respectively. In LED-treated, 100% inhibition was detected for both SO-AgNP and TV-AgNP at concentrations 25 and 50 mg/L. Mala et al. [36] examined the application of antimicrobial photodynamic inactivation against *S. aureus* and *K. pneumoniae* using photosensitizers when AgNPs were combined with porphyrin. In conclusion, they suggested that combining AgNPs treatment and antimicrobial photodynamic inactivation may be a promising approach to treating bacterial infection. As a result of the present study, the use of SO-AgNP and TV-AgNP in combination with light led to a decrease in the effect of *E. coli* cell viability; it can help fight microorganisms in the treatment of infectious diseases and increase the efficiency of APDT.

### 3.11. Biofilm inhibition activity

**Figure 12:**
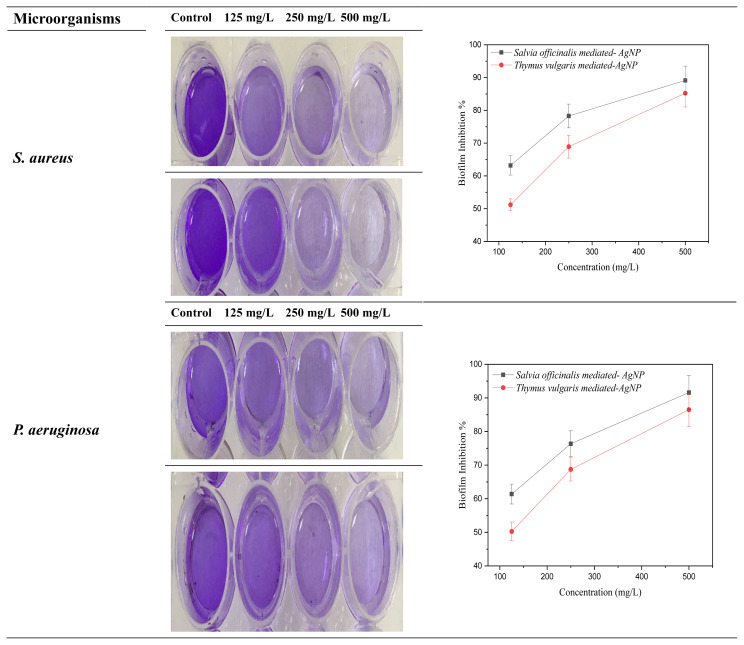
Biofilm inhibition of SO-AgNP and TV-AgNP.

The antibiofilm efficacy of SO-AgNP and TV-AgNP on the development of biofilm *P. aeruginosa* and *S. aureus* was confirmed through crystal violet assay. The results of biofilm inhibition are shown in Figure 12. *P. aeruginosa* and *S. aureus* were applied to investigate the inhibition activity of biofilm. The biofilm inhibition results of *S. aureus* and *P. aeruginosa* for SO-AgNP was more effective than TV-AgNP. The inhibition activity results of biofilm against *S. aureus* were determined as 63.18%, 78.29%, 89.14% for SO-AgNP and 51.19%, 68.92%, 85.23% for TV-AgNP at 125 mg/L, 250 mg/L and 500 mg/L, respectively.

The biofilm inhibiting efficiency against *P. aeruginosa* was also determined around 61.39%, 76.35%, 91.58% for SO-AgNP and 50.27%, 68.73%, 86.49% for TV-AgNP, at 125 mg/L, 250 mg/L and 500 mg/L, respectively. Srinivasan et al. [37] biogenically synthesized Ag nanoparticles through *Piper betle* aqueous removal and evaluated the antibiofilm activity versus *S. marcescens* and *P. mirabilis*. They reported 71%–63% and 69%–53% biofilm inhibition for two different strains of *S. marcescens* and *P. mirabili*s, respectively. Porter et al. [38] studied the antibiofilm activity of AgNPs containing glass ionomer types of cement, and they reported that AgNP-modified Fuji IX indicated the maximum reduction in biofilm formation by 99%. Lara et al. [15] indicated that Ag nanoparticles blocked biofilm creation of *Candida auris* in both medical and environmental samples. AgNPs and ions related to these particles show antimicrobial activity by damaging the structure of biofilm and ingredients and preventing the bacterial metabolism by different procedures. As exerting antimicrobial activity, AgNPs are considered to engage the biofilm, penetrate, migrate internally, and interact with key constituents of the biofilm such as lipids, nucleic acids, proteins, and polysaccharides via hydrophobic, electrostatic, Van der Waals, ionic interactions, hydrogen bonding [39]. According to the biofilm inhibition results, a high reduction in biofilm formation was monitored in wells treated with green synthesized Ag nanoparticles compared to control wells. Therefore, the present study shows green synthesized AgNPs as a possible choice against the classic antibiotics in checking biofilm-related bacterial contaminations.

The nanoparticles of noble metals such as Ag, Au, Pd, and Pt are widely used due to their unique characteristics such as conductivity and high stability. Silver nanoparticles (AgNPs) can be produced in different ways besides green synthesis, such as physical, chemical reduction, sonochemical, and microwave-assisted. However, the biological synthesis of AgNPs is a low-cost and the safest alternative approach and synthesis route as it does not require harmful reagents, which makes it less toxic and environmentally friendly. Promising nanomaterial production, which researchers focus on, is expected to have many uses such as clean environment, energy storage tools, durable material production, dirt-repellent clothing, and diagnosis and treatment of diseases. The use of silver particles in bandages for quick healing of wounds indicates that it can also be used in many creams and medicines. It is thought that the biological properties of SO-AgNP and TV-AgNP differ in some parameters depending on the particle size and the interaction area.

## 4. Conclusion

In this present study, silver nanoparticles (AgNPs) were green synthesized from *Salvia officinalis* and *Thymus vulgaris*, called phytotherapy plants. The produced products were analyzed using SEM, FTIR, XRD, and UV-Vis spectroscopy techniques. For green synthesized SO-AgNP and TV-AgNP, a couple of biological properties such as antioxidant, DNA cleavage, biofilm inhibition activity, antimicrobial photodynamic therapy, microbial cell viability, and antimicrobial activity were also examined. The obtained results verified that the antioxidant activities of the synthesized AgNPs were in a concentration-dependent mode. These studies proved that the green synthesis silver nanoparticles had good antimicrobial and biofilm inhibition activities. The agarose gel electrophoresis study was performed to determine single-strand DNA cleavage activities of SO-AgNp and TV-AgNp. It was seen that SO-AgNp and TV-AgNp had single-strand DNA cleavage activity. The findings demonstrated that SO-AgNp and TV-AgNp exhibited 100% cell viability inhibition and it was found that they showed slightly antimicrobial photodynamic activity. As a conclusion, the current study indicated that using ecofriendly green-synthesized AgNPs can find application soon in biomedical drug and nanobiotechnology.

## SUPPLEMENTARY INFORMATION

### DPPH activity

The antioxidant activities of SO-AgNP and TV-AgNP were conducted by determining the scavenging activity of DPPH according to the technique reported by Ağırtaş et al. [1] by some revisions. In brief, 250 μL of five various concentrations (12.5–200 mg/mL) of SO-AgNP, TV-AgNP, standard ascorbic acid, and Trolox were taken in different test tubes. Next, 1000 μL of 1-diphenyl-2-picrylhydrazyl methanol solution was added to the above samples, vortexed thoroughly, and incubated for 30 min in a dark place. Following the 30-min incubation, the scavenge efficiency was detected with UV/Vis spectrophotometer at 517 nm, and radical scavenging activity was computed using the following formula (1):


(1) $$Capacity\;(\%)=\left(\frac{Abs(control)-Abs(sample)}{Abs(control)}\right)\times 100$$

where *Abs_control_* is the checking absorbance and *Abs_sample_* is the absorbance data of studied compounds and DPPH after 30 min of incubation.

### The study of ferrous ion chelating efficiency

The ferrous ion chelating capability of SO-AgNP and TV-AgNP was evaluated with the Dinis method [2]. The SO-AgNP and TV-AgNP at concentrations ranging from 25 to 200 mg/L were interacted to FeCl_2_ for 2 min, and later ferrozine was put in. After that, the reaction solution was incubated for 10 min in a dark place, and then ferrous ion chelating capability was detected by UV/Vis spectra. The absorption spectra data of the reaction mixtures were recorded at 562 nm and the percent of chelating ability was computed using the formula (2):


(2) $$Metal\;Chelating\;Effect\;(\%)=\left(\frac{Abs(control)-Abs(sample)}{Abs(control)}\right)\times 100$$

where the absorbance of checking reaction is *Abs_control_*, and *Abs_sample_* indicates the absorption spectra of produced in the presence of the compounds or EDTA.

### The study of antimicrobial property

The antibacterial activity of the SO-AgNP and TV-AgNP was detected by measuring their MICs via the microdilution method. The tested microbial strains were *Enterococcus faecalis, Staphylococcus aureus*, *Enterococcus hirae*, *Escherichia coli, Pseudomonas aeruginosa*, *Legionella pneumophila subsp. Candida tropicalis*, *pneumophila*, *and Candida parapisilosis*. The MICs were investigated using 96-well microtiter plates. The testing microorganisms were grown for 18 h before dilution. The serial dilutions of AgNPs were performed and then microbial strains prepared with 0.5 McFarland Scale were put into the microplate wells. Next, the plates were put in an oven for a 24-h incubation at 37 °C. After 24 h of incubation, antimicrobial activity was determined via the values of MIC specified as the lowest amount that restrained the microbial upgrowth.

### The study of DNA cleavage

DNA cleavage properties of the newly synthesized SO-AgNP and TV-AgNP were investigated with agarose gel electrophoresis using pBR322 plasmid DNA as a target. Different concentrations of SO-AgNP and TV-AgNP were mixed with the plasmid DNA and later the sample was incubated at 37 °C for 60 min. Afterward, the mixtures were loaded into wells of agarose gel. Later, the electrophoresis process was performed at a voltage of 80 V for 60 min. The untreated genomic DNA was used as a negative control. The bands of DNA were visualized via a transilluminator.

### Testing of bacterial viability

In this study, ATCC 10536 (*E. coli)* was used as a bacterial strain to investigate the cell viability inhibition properties of SO-AgNP and TV-AgNP. Afterwards, the microorganism was implanted into Nutrient Broth ambient, the mixture was allowed to incubate for 24 h at 37 °C at 150 rpm in a shaker. After 24 h of incubation, the sample was centrifuged for 5 min at 5000 rpm. The obtained microbial pellet was cleaned using a sterile saline solution to separate the culture ambience residual. The cleaned ATCC 10536 was transferred into 10 mL solution of NaCl. The microbial mixture was used for the cell viability test. *E. coli* was allowed to react with SO-AgNP and TV-AgNP prepared at three different concentrations (125, 250, and 500 mg/L) for 90 min at 37 °C. At the end of the period, the solution mixtures were diluted into different fractions, infused in solid NB medium, and then were left to incubate at 37 °C for 24 h. The process was also performed for the control group that did not contain AgNPs. In the end, the colonies were counted, and the cell viability was estimated with the formula (3).


(3) $$\%\;Cell\;viability:[(A_{control}-A_{sample})/A_{control}]\times 100.$$

### Antimicrobial photodynamic therapy by AgNPs

*E. coli* was prepared according to the cell viability study mentioned above. SO-AgNP and TV-AgNP at 50 mg/L concentration were incubated with *E. coli* in the dark place at 25 °C for 15 min and then exposed to LED for 5 min. After irradiation, the mixtures were diluted in different fractions. After the dilution step, inoculation was made in solid NB medium; afterwards, the sample was left to incubation at 37 °C for 24 h. A similar process was also carried out for the control group that did not contain SO-AgNP and TV-AgNP. Then the colonies were counted, and the antimicrobial photodynamic therapy by AgNPs was calculated using equation (3).

### Biofilm inhibition activity

The effects of newly synthesized SO-AgNP and TV-AgNP on *P. aeruginosa* and *S. aureus* biofilm formation were analyzed by the crystal violet (CV) assay in 24-well plates. 24-well plates containing different concentrations of AgNPs were inoculated with *S. aureus* and *P. aeruginosa* and left to incubation at 37 °C for 72 h. When the incubation period was over, the plate’s wells were softly drained and then cleaned twice using distilled water. These plates were left to dry for 30 min in an oven set at 60 °C. Later, CV was added to the wells to paint the biofilms for 45 min. The crystal violet was then removed and the plates were bathed gently. The same bathing process was performed twice. Afterwards, ethanol solution was added and allowed to incubate for 15 min to recover the absorbed CV. A spectrophotometer determined the inhibiting of biofilm at around 595 nm. The wells comprising only *S. aureus* and *P. aeruginosa* with medium were utilized as the positive controls. The value of biofilm inhibition was calculated with the formula (4).


(4) $$Biofilm\;Inhibition\;(\%)=\left(\frac{Abs(control)-Abs(sample)}{Abs(control)}\right)\times 100$$

References1

AgırtaşMS
KaratasC
ÖzdemirS

Synthesis of some metallophthalocyanines with dimethyl 5- (phenoxy) -isophthalate substituents and evaluation of their antioxidant-antibacterial activities
Spectrochimica Acta Part A: Molecular and Biomolecular Spectroscopy
135
2015
20
24
2503664510.1016/j.saa.2014.06.1392

DinisTCP
MadeiraVMC
AlmeidaLM

Action of phenolic derivatives (acetaminophen, salicylate, and 5-aminosalicylate) as inhibitors of membrane lipid peroxidation and as peroxyl radical scavengers, Arch
Biochem Biophys
315
1994
161
169
10.1006/abbi.1994.14857979394

## Figures and Tables

**Table:** The minimum inhibition concentration (MIC) of test microorganisms.

Microorganisms	SO-AgNp	TV-AgNp
*E. coli*	256	512
*P. aeruginosa*	256	512
*L. pnumophila* subsp. *pneumophila*	256	512
*E. hirae*	128	256
*E. fecalis*	32	64
*S. aureus*	128	256
*C. parapisilosis*	256	512
*C. tropicalis*	128	256

* mg/L
